# Reactive Oxygen Species Generation-Scavenging and Signaling during Plant-Arbuscular Mycorrhizal and *Piriformospora indica* Interaction under Stress Condition

**DOI:** 10.3389/fpls.2016.01574

**Published:** 2016-10-21

**Authors:** Manoj Nath, Deepesh Bhatt, Ram Prasad, Sarvajeet S. Gill, Naser A. Anjum, Narendra Tuteja

**Affiliations:** ^1^Amity Institute of Microbial Technology, Amity University Uttar PradeshNoida, India; ^2^Department of Biotechnology, Shree Ramkrishna Institute of Computer Education and Applied Sciences, Veer Narmad South Gujarat UniversitySurat, India; ^3^Stress Physiology and Molecular Biology Laboratory, Centre for Biotechnology, Maharshi Dayanand UniversityRohtak, India; ^4^Centre for Environmental and Marine Studies and Department of Chemistry, University of AveiroAveiro, Portugal

**Keywords:** plant root, ROS-metabolism, ROS-signaling, stress, arbuscular mycorrhizal fungi

## Abstract

A defined balance between the generation and scavenging of reactive oxygen species (ROS) is essential to utilize ROS as an adaptive defense response of plants under biotic and abiotic stress conditions. Moreover, ROS are not only a major determinant of stress response but also act as signaling molecule that regulates various cellular processes including plant-microbe interaction. In particular, rhizosphere constitutes the biologically dynamic zone for plant–microbe interactions which forms a mutual link leading to reciprocal signaling in both the partners. Among plant–microbe interactions, symbiotic associations of arbuscular mycorrhizal fungi (AMF) and arbuscular mycorrhizal-like fungus especially *Piriformospora indica* with plants are well known to improve plant growth by alleviating the stress-impacts and consequently enhance the plant fitness. AMF and *P. indica* colonization mainly enhances ROS-metabolism, maintains ROS-homeostasis, and thereby averts higher ROS-level accrued inhibition in plant cellular processes and plant growth and survival under stressful environments. This article summarizes the major outcomes of the recent reports on the ROS-generation, scavenging and signaling in biotic-abiotic stressed plants with AMF and *P. indica* colonization. Overall, a detailed exploration of ROS-signature kinetics during plant-AMF/*P. indica* interaction can help in designing innovative strategies for improving plant health and productivity under stress conditions.

## Introduction

Plant–microbe interactions cover a broad range of relationships between plant and microbial community in which either of the partners participate by imposing a beneficial, negative or neutral effect on its counterpart. Moreover, plant roots are continuously exposed to a large number of microbes present in the rhizosphere that influence plant life cycle and overall fitness (Sanders, 2011; Mine et al., 2014). Plant–microbe symbiotic interactions have been the focus of recent plant stress research, where the outcomes of these interactions were credibly evidenced to alleviate biotic and abiotic stress-impacts and consequently enhance the plant fitness (Goh et al., 2013; Schouteden et al., 2015; Doty, 2016). In the present scenario, a relatively small number of beneficial plant-microbe interactions are well characterized and utilized (Farrar et al., 2014). Microbial counterpart- arbuscular mycorrhizal fungi (AMF)-mediated stress tolerance and growth enhancements have been extensively reported in colonized host plants during symbiotic interaction studies (Muthukumar and Udaiyan, 2010; Porcel et al., 2012; Tahat and Sijam, 2012). Notably, a number of recent works have discussed the significance of *Piriformospora indica*, a arbuscular mycorrhizal-like fungi which is able to grow in pure culture and without the presence of the plant. *P. indica*, a multifunctional and versatile root endophytic fungus belongs to Sebacinales (order-Basidiomycota) and is involved in the improvement of growth, yield, and plant tolerance to major biotic and abiotic stresses (Sherameti et al., 2008; Vadassery et al., 2009a,b; Cruz et al., 2013; Jogawat et al., 2013; Prasad et al., 2013; Bakshi et al., 2014; Johnson et al., 2014; Gill et al., 2016; Trivedi et al., 2016). Both fungal counterparts viz., AMF and *P. indica* are capable of improving plant fitness via changing mainly the chemical plasticity through altering reactive oxygen species (ROS) generation-scavenging under biotic and abiotic stresses (Beneventi et al., 2013; Goh et al., 2013; Hashem et al., 2016; Mo et al., 2016). ROS can be both radical and non-radical forms and generated in normal metabolic processes e.g., as a result of electron transport chains in chloroplast and mitochondria. However, adverse conditions including abiotic and biotic stresses can significantly accelerate the generation of ROS at cellular level (Apel and Hirt, 2004; Gill and Tuteja, 2010; Rasool et al., 2013). Radical forms of ROS majorly include superoxide radicals ($O_{2}^{•–}$), perhydroxy radical (${\mathrm{HO}}_{2}^{•}$) and alkoxy radicals (RO); whereas, hydrogen peroxide (H_2_O_2_) and singlet oxygen (^1^O_2_) are included in non-radical molecular form. Compared with non-radicals, radical forms of ROS are more toxic due to their highly reactive nature (Gill and Tuteja, 2010; Sewelam et al., 2016). In plants, enzymatic and non-enzymatic systems are the two major components of ROS-scavenging system. The list of major enzymatic components includes superoxide dismutase (SOD), ascorbate peroxidase (APX), monodehydroascorbate reductase (MDHAR), guaiacol peroxidase (GPX), glutathione reductase (GR), peroxidase (POD), and catalase (CAT). Major antioxidant metabolites namely glutathione (GSH) and ascorbic acid (AsA) belong to the list of non-enzymatic component (Apel and Hirt, 2004; Gill and Tuteja, 2010; Rasool et al., 2013). Notably, NADPH oxidases and respiratory burst oxidase homologs are the key components of ROS generation system in plants (Suzuki et al., 2013; Kadota et al., 2015).

In order to alert the plants for stress-adaptation, initial generation of ROS was reported to act as long distance signals in response to stress (Mittler et al., 2011; Sewelam et al., 2016). Furthermore, ROS are also thought to be generated during early stages of symbiotic interactions of mycorrhizal fungi associated with plant roots (Fester and Hause, 2005; Tanaka et al., 2006; Puppo et al., 2013; Espinosa et al., 2014; Kiirika et al., 2014). Though, to efficiently utilize ROS as signaling molecule, plants must sustain a precise balance between ROS generation and ROS-scavenging pathways in order to finally mitigate the potential toxic effects of ROS (Mittler et al., 2004; Baxter et al., 2014). In plants, stress signals include redox homeostasis, antioxidants signaling and continuous production/scavenging of ROS at cellular level (Bose et al., 2014; Jajic et al., 2015). However, severity or prolonged duration of biotic and abiotic stresses can reduce the capability of plant to neutralize excess ROS production that alternatively cause oxidative stress and finally affect cellular essential metabolic activities and viability (Gill and Tuteja, 2010; Barna et al., 2012; Nath et al., 2016).

Despite the previous facts, literature is scanty on how the generation, signaling and metabolism of ROS can be modulated in plants with AMF/*P. indica* association under stress conditions. Hence, this paper aims to briefly appraise ROS accumulation, homeostasis, and signaling during plant-AMF and *P. indica* interaction in response to major stress conditions.

## ROS Generation and Scavenging During Plant-Arbuscular Mycorrhizal Interaction Under Stress Conditions

Reactive oxygen species profiling in AMF-inoculated roots of several plants including *Medicago truncatula, Zea mays*, and *Nicotiana tabacum* has evidenced important role of mycorrhizal colonization/arbuscules in the scavenging of major ROS such as H_2_O_2_ (Fester and Hause, 2005). AMF-colonization improved drought tolerance in olive plants, where compared to non-colonized olive plants, AMF-colonized plants exhibited lesser accumulation of ROS (H_2_O_2_) and malondialdehyde (MDA), a lipid peroxidation product (Fouad et al., 2014). Similar results were also reported in other test plants including date palm (Benhiba et al., 2015) and *Citrus reticulata* (Sarkar et al., 2016), where improved drought tolerance was dedicated to AMF-mediated improvements in the antioxidant defense of host plants and alleviate drought stress-effects. A recent report also confirmed the role of AMF (*Glomus versiforme*) colonization in the enhancement of ROS-metabolism via its modulatory role in the activities of antioxidant enzymes including SOD, CAT, APX, GR, and MDHAR in drought stressed water melon plants (Mo et al., 2016). AMFs colonization-mediated strengthening of antioxidants defense systems was advocated to control ROS-metabolism and eventually alleviate oxidative stress in host plants under stress conditions (Peterson et al., 2004; Vos et al., 2013; Wu et al., 2014; Hashem et al., 2016). Involvement of ROS generation was also suggested in providing resistance in soybean against nematode (*Meloidogyne javanica*) infection (Beneventi et al., 2013). In mycorrhizal tomato roots, reduction of infection caused by root-knot nematode (*M. incognita)* was linked with ROS metabolism (Vos et al., 2013). Enhanced activities of major antioxidant enzymes including SOD, CAT, POD, GR, and APX were argued to improve cadmium (Cd)-tolerance in tomato via AMF-mediated ROS-scavenging (Hashem et al., 2016). **Table 1** summarizes representative studies highlighting plant-AMF/AMF-like (*P. indica*) interaction and its link with ROS metabolism in response to various biotic and abiotic stress conditions.

**Table 1:** Representative studies highlighting AMF/*P. indica* mediated-stress tolerance associated with the metabolism of reactive oxygen species (ROS) in different plants.

Name of the interacting fungi	Plant	Stress tolerance	ROS metabolism in colonized plants	^∗^Potential stress tolerance-mechanism	Reference
*Glomus mosseae*	*Solanum lycopersicum* (Tomato)	Cadmium (Cd) stress	Increased level of SOD, CAT, POD, GR, and APX	AMF-mediated ROS scavenging	Hashem et al., 2016
*G. mosseae*	*Medicago sativa* (alfalfa)	Atrazine (Herbicide) stress	High level of thioredoxin, glutaredoxin and GPX	High GPX activity may link with alleviation of atrazine stress	Song et al., 2016
*Piriformospora indica*	*Hordeum vulgare* (Barley)	Salt stress	High antioxidant activities and glutathione-ascorbate cycle activation	Stress tolerance link with increase in antioxidants	Waller et al., 2005; Baltruschat et al., 2008
		Biotic stress (*Fusarium culmorum*)	Increased antioxidants	Stress tolerance link with increase in ROS metabolism	Waller et al., 2005
	*Zea mays* (Maize)	Biotic stress (*Fusarium verticillioides)*	High antioxidant enzymatic activities	High antioxidants proposed to link with stress tolerance	Kumar et al., 2009
*P. indica* and *Azotobacter chroococcum* (Co-inoculation)	*Triticum aestivum* (Wheat)	Zinc stress	High APX and peroxidase activity	Induced antioxidant activities	Abadi and Sepehri, 2016
*Rhizophagus manihotis and Funneliformis mosseae*	*Olea europaea* (Olive)	Drought stress	Low H_2_O_2_ in AMF-colonized plants	Low H_2_O_2_ level correlated with drought tolerance	Fouad et al., 2014
*R. intraradices and F. mosseae*	*Phoenix dactylifera* (Date Palm)	Drought stress	High antioxidant- enzymatic activities	Antioxidant defense system alleviates long term drought stress.	Benhiba et al., 2015
*Glomus* sps.	*Citrus reticulate* (Mandarin orange)	Drought stress	High antioxidant- enzymatic activities	Increased antioxidant defense system link with oxidative stress tolerance	Sarkar et al., 2016
*G. mosseae*	*S. lycopersicum* (Tomato)	biotic stress (*Meloidogyne incognita*)	Reduction of root-knot nematode infection	Involvement of ROS metabolism with reduction of the nematode infection	Vos et al., 2013

*^∗^ROS may be one of the associated mechanisms or it may likely to have link with stress tolerance.*

## Link of ROS Signaling with Stress Tolerance During Plant-Arbuscular Mycorrhizal Association

In order to adapt with various biotic and abiotic stresses, plants are endowed with a highly complicated and elaborated signaling cascade. In response to stress conditions, plants utilize ROS as one of the key signaling players which also activate other defense related signaling pathways (Baxter et al., 2014; Xu and Brosche, 2014; Sewelam et al., 2016). Transcriptome analysis of *Glomus mosseae/Medicago sativa* during herbicide (atrazine) stress revealed higher stress tolerance via increased expression of electron transport related genes, ROS-scavenging antioxidants such as thioredoxin, glutaredoxin, and GPX. Additionally, a higher degradation of atrazine was also observed in mycorrhizal (*G. mosseae*)-treated *M. sativa* plants (versus non-treated plants), further corroborated its link with stress mitigation (Song et al., 2016).

Increasing evidences revealed that ROS-generation is one of the most frequent responses triggered in plants that represent a general point for different signaling cascades under stress (Sewelam et al., 2016). ROS generation is also one of the characteristics of the early host-defense system during initial microbial invasion with host plants and can also lead to the hypersensitive reaction and cell death at the site of interaction (Puppo et al., 2013). However, detailed reports on ROS signature kinetics are still very limited during initial stages of microbial interaction with plant. A transient increase of ROS was observed within seconds in root hairs of *Phaseolus vulgaris* after treatment with Nod factors (NFs), where specific role of ROS response during symbiotic association was proposed (Cardenas and Quinto, 2008). Moreover, among ROS, H_2_O_2_ is membrane-permeable and plays an important role in signaling cascade as well as in defense response under stressful environments (Xia et al., 2009; Saxena et al., 2016). Thus, H_2_O_2_ has emerged as an active signaling player which is also involved in regulation of specific biological reactions/cellular metabolism and stress tolerance (Neill et al., 2002; Yan et al., 2007; Saxena et al., 2016). In *M. truncatula*–*Sinorhizobium meliloti*, exogenously supplied H_2_O_2_ was associated with induced *MtSpk1* gene (encoding a putative protein kinase) and also its conformed functional role was argued in the control of genes linked to rhizobia symbiosis (Andrio et al., 2013).

## ROS Modulation During Interaction of Plants and *P. indica* Under Stress Condition

*Piriformospora indica* mediated stress tolerance has been credibly reported in various crops including barley (Waller et al., 2005; Deshmukh and Kogel, 2007), wheat (Serfling et al., 2007), maize (Kumar et al., 2009), tomato (Sarma et al., 2011), and lentil (Dolatabadi et al., 2012). In rhizosphere, *P. indica* was reported to enhance the levels of alkaline phosphatase and acid phosphatase enzymes that in turn contributes for higher phosphate uptake in plants (Das et al., 2014). However, information is still meager on the relation of *P. indica* with the status of ROS in plants with mycorrhizal association. Nevertheless, the control of ROS generation and the modulation of major components of antioxidant defense pathway were argued as a key mechanism underlying *P. indica* mediated improved stress tolerance in wheat, barley and maize (Waller et al., 2005; Serfling et al., 2007; Kumar et al., 2009). In plant roots, ROS generation and activation of defense related responses was reported during initial mycorrhizal associations (Pozo and Azcón-Aguilar, 2007). Notably, the generation of ROS was initially observed before physical contact of *P. indica* with plant roots and no H_2_O_2_ was reported after establishment of symbiotic relationship between *P. indica* and plant root (Vadassery et al., 2009a; Camehl et al., 2011; Vahabi et al., 2015).

H_2_O_2_ was found to induce *OXI1* (*Oxidative Signal Inducible1*) gene which consequently triggers defense response during pathogen infection (Rentel et al., 2004; Anthony et al., 2006; Petersen et al., 2009). In *Arabidopsis* roots, *OXI1* (a serine/threonine kinase) was shown to be required for oxidative burst/ROS-mediated responses including root hair elongation and disease tolerance against biotrophic pathogens (Rentel et al., 2004; Petersen et al., 2009). Though, under favorable co-cultivation conditions, H_2_O_2_ generation was repressed in *P. indica*-colonized *Arabidopsis* roots while stimulation of growth response via *P. indica* involved PLD-PDK1-OXI1 cascade in *Arabidopsis* (Camehl et al., 2011). Activation of the GSH-AsA cycle followed by increased antioxidant capacity was reported in *P. indica* colonized barley root (Waller et al., 2005). *P. indica*-mediated enhancement of antioxidants was reported to link with salt stress tolerance in the colonized barley plants (Baltruschat et al., 2008). Microbe derived effectors delivered during plant-mycorrhizal association can enhance the microbial infections and also manipulate the host metabolism. Recently, a study demonstrated that the expression of candidate effector (PIIN_08944) of *P. indica* was found to decrease the ROS burst activated by flg22 and chitin in barley (Akum et al., 2015). Co-inoculation of *P. indica* and *Azotobacter chroococcum* in wheat enhanced APX and peroxidase-antioxidant enzyme activities under zinc-deprived environment (Abadi and Sepehri, 2016).

**FIGURE 1:**
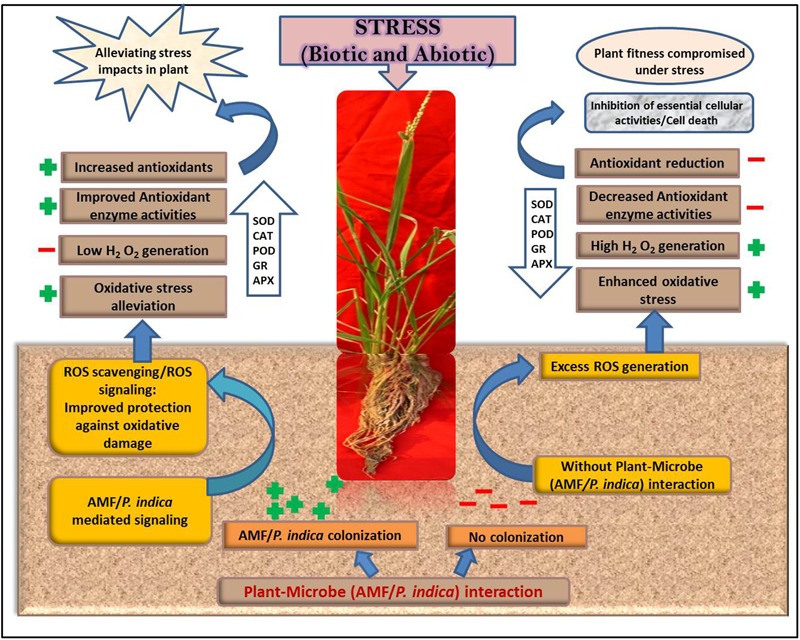
**Schematic representation of reactive oxygen species (ROS) generation and signaling during plant- arbuscular mycorrhizal fungi (AMF)/*Piriformospora indica* interaction in response to stress.** The left panel of the figure indicates the ROS generation and signaling in presence of AMF/*P. indica* interaction, while the right panel demonstrates high ROS in absence of mycorrhizal interaction. AMF/*P. indica* colonization in plant roots can help the plant to cop the detrimental effect of stress, directly or indirectly, on plant functionality and metabolism. Altered ROS signaling/metabolism, in response to biotic and abiotic stress, link with stress tolerance in mycorrhizal colonized plants consequently provides stress tolerance; while, the scenario is just reverse in case of non-colonized plants i.e., high ROS production followed by the inhibition of plant cellular activities thus affecting the plant fitness. AMF/*P. indica* colonized plants were able to withstand stress induced damage by increasing the production of various antioxidant compounds, which helps to scavenge ROS and thus in turn enhance the activities of various antioxidant enzymes as listed inside the arrow. The positive (+) and negative (-) sign in figure denotes an increased and decreased levels, respectively. Superoxide dismutase (SOD), Catalase (CAT). Peroxidase (POD), Glutathione reductase (GR), and Ascorbate peroxidase (APX).

Recently, the exudates released via *P. indica* interaction were reported to initially lead to ROS generation, accumulation of stress-responsive phytohormone, stomatal closure and induce the defense responsive genes in root and/or shoot of *Arabidopsis*. Moreover, after the establishment of physical contact of plant with *P. indica*, defense responsive genes expression/number, phytohormone and ROS levels turned down; whereas, the stomata re-opened (Vahabi et al., 2015). **Figure 1** schematically highlights the link of ROS generation, scavenging and signaling with plant-mycorrhizal association and their cumulative effect on the enhanced plant fitness under stress.

## Conclusions and Perspectives

Symbiotic microbial association can enhance the ROS-antioxidant defense system and ultimately improve the plant fitness under stress. Further, in future, exploration of ROS signatures kinetics during initial plant-arbuscular mycorrhizal association can enhance the basic understanding of mycorrhizal link with ROS generation. Additionally, molecular insights into the detailed kinetics of ROS metabolism in plant-mycorrhizal especially *P. indica* signaling are advocated to design innovative strategies via modulating the ROS metabolism and ultimately will help to improve plant productivity under stress conditions.

## Author Contributions

MN and NT developed the idea and wrote/finalized the MS. DB, RP, SG, and NA made the figures and developed table and helped in writing. All authors read and approved the final manuscript.

## Conflict of Interest Statement

The authors declare that the research was conducted in the absence of any commercial or financial relationships that could be construed as a potential conflict of interest.
